# Development and validation of a machine learning-based detection system to improve precision screening for medication errors in the neonatal intensive care unit

**DOI:** 10.3389/fphar.2023.1151560

**Published:** 2023-04-14

**Authors:** Nadir Yalçın, Merve Kaşıkcı, Hasan Tolga Çelik, Karel Allegaert, Kutay Demirkan, Şule Yiğit, Murat Yurdakök

**Affiliations:** ^1^ Department of Clinical Pharmacy, Faculty of Pharmacy, Hacettepe University, Ankara, Türkiye; ^2^ Department of Biostatistics, Faculty of Medicine, Hacettepe University, Ankara, Türkiye; ^3^ Division of Neonatology, Department of Child Health and Diseases, Faculty of Medicine, Hacettepe University, Ankara, Türkiye; ^4^ Department of Pharmaceutical and Pharmacological Sciences, KU Leuven, Belgium; ^5^ Department of Development and Regeneration, KU Leuven, Belgium; ^6^ Department of Hospital Pharmacy, Erasmus Medical Center, Rotterdam, Netherlands

**Keywords:** newborn, medication error, machine learning, drug safety, clinical pharmacy, data collection, adverse drug reaction

## Abstract

**Aim:** To develop models that predict the presence of medication errors (MEs) (prescription, preparation, administration, and monitoring) using machine learning in NICU patients.

**Design:** Prospective, observational cohort study randomized with machine learning (ML) algorithms.

**Setting:** A 22-bed capacity NICU in Ankara, Turkey, between February 2020 and July 2021.

**Results:** A total of 11,908 medication orders (28.9 orders/patient) for 412 NICU patients (5.53 drugs/patient/day) who received 2,280 prescriptions over 32,925 patient days were analyzed. At least one physician-related ME and nurse-related ME were found in 174 (42.2%) and 235 (57.0%) of the patients, respectively. The parameters that had the highest correlation with ME occurrence and subsequently included in the model were: total number of drugs, anti-infective drugs, nervous system drugs, 5-min APGAR score, postnatal age, alimentary tract and metabolism drugs, and respiratory system drugs as patient-related parameters, and weekly working hours of nurses, weekly working hours of physicians, and number of nurses’ monthly shifts as care provider-related parameters. The obtained model showed high performance to predict ME (AUC: 0.920; 95% CI: 0.876–0.970) presence and is accessible online (http://softmed.hacettepe.edu.tr/NEO-DEER_Medication_Error/).

**Conclusion:** This is the first developed and validated model to predict the presence of ME using work environment and pharmacotherapy parameters with high-performance ML algorithms in NICU patients. This approach and the current model hold the promise of implementation of targeted/precision screening to prevent MEs in neonates.

**Clinical Trial Registration:** ClinicalTrials.gov, identifier NCT04899960.

## Highlights

• Medication errors (MEs) are quite common in newborns admitted to the neonatal intensive care unit, and risk factors make it difficult to optimize their pharmacotherapy and to prevent MEs.
• The most important variables in predicting the presence of ME were the total number of drugs and the prescription of anti-infective drugs.
• The model predicting the presence of physician- or nurse-related MEs correctly classified 92.0% of the patients.
• Machine learning can be instrumental to implement precision screening on MEs in the neonatal intensive care setting.
• This high-performance prediction model can be used to compensate for this increasing workload and decreasing the number of qualified healthcare providers.

## 1 Introduction

At least one medication error (ME) occurs in 74.8% of neonates admitted to the neonatal intensive care unit (NICU). The most commonly reported MEs in neonates relate to “wrong dose” (28%) or “wrong administration” (29%) (Eslami et al., 2019). Since (patho) physiological changes such as weight or body composition evolve rapidly in neonates over time (“time-dependent physiology”), the pharmacokinetics and pharmacodynamics of any drug are also likely to change. In addition to these changes, kidney and liver dysfunction, immunodeficiency, and susceptibility to infection are also commonly observed in the NICU. Due to the subsequent constantly evolving clinical needs, frequent changes in the type, dose, and number of drugs are common. Such factors make it difficult to optimize their pharmacotherapy and to prevent MEs (Allegaert and Sherwin, 2016).

MEs may occur during prescription (14%–74%), preparation (11.9%–25%), administration (31%–63%), or monitoring (1.4%) process in neonates and are more common in neonates than in children and adolescents (Kaushal et al., 2001; Kunac et al., 2009). It is assumed that the higher prevalence of these MEs in hospitalized neonates is related to the diversity of drugs and doses used and differences in postnatal age, gestational age, birth weight, or diagnoses.

As the World Health Organization declared MEs as a global patient safety problem, a goal to reduce the severe avoidable harms associated with MEs by 50% within the next 5 years has been set. Related to the NICU, it is claimed that half of the MEs can be prevented (Sakuma et al., 2014).

To operationalize this, we aimed to develop approaches and models that predict MEs detected by the clinical pharmacist throughout the pharmacotherapy process (prescription, preparation, administration, and monitoring) of the patients admitted to the NICU with a newborn-centered approach, using artificial intelligence (machine learning algorithms). In doing so, the intention is to reduce the workload of physicians and nurses while preventing MEs as part of pharmacotherapy optimization.

## 2 Materials and methods

### 2.1 Study design and population

**FIGURE 1:**
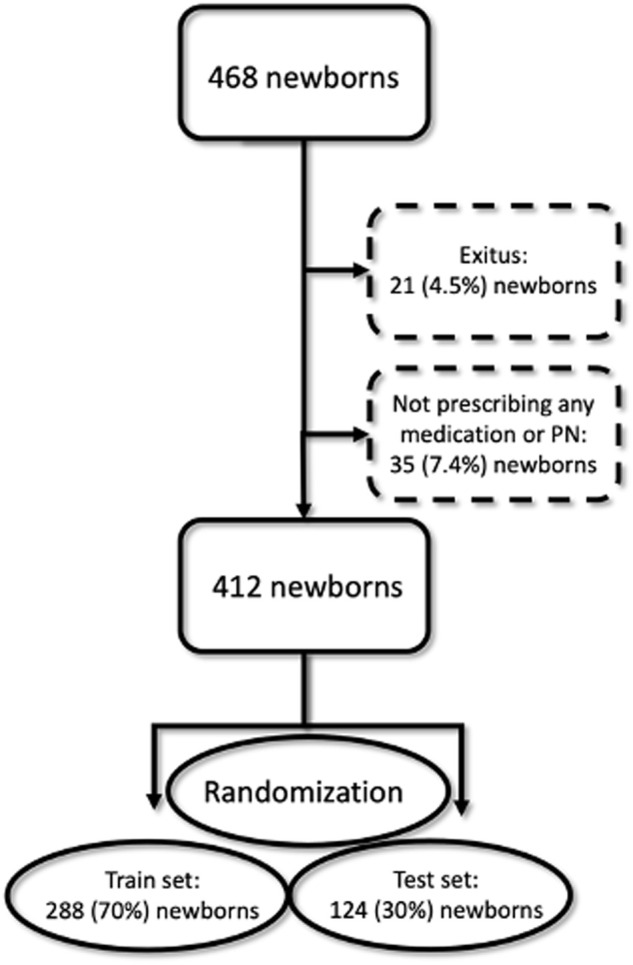
Flow chart of machine learning procedure.

This prospective cohort study randomized with 10-fold cross validation in machine learning was conducted between February 2020 and June 2021 in a NICU with 22-bed capacity. The study was conducted as a single-center analysis at Hacettepe University Hospital. Although there is no exact sample size calculation for machine learning-based prediction models, we assumed a maximum of 10 independent variables in the final model and aimed to get at least 20 events per independent variable. All admitted neonates to whom at least one systemic drug was prescribed were included during the 17-month data collection period. A flowchart about the participants is provided in Figure 1.

### 2.2 Data acquisition and ME detection

Patients’ follow-up was performed daily to assess the clinical characteristics and medical conditions by a comprehensive assessment using *Micromedex Neofax* and *UpToDate* as reference databases of drug information. Demographic, clinical, and MEs in terms of prescription (wrong drug, unit, dose, dose interval, infusion rate, and diluent), preparation (wrong drug, occupational safety, and storage), administration (omission, extra dose, wrong time, infusion, and technique), and monitoring (physical, vital, laboratory, and therapeutic drug monitoring) data were obtained from prospectively routine follow-up. For the quantitative parameters (dose, time, infusion rate, etc.), those with a margin of errors more than 5% were accepted as MEs.

### 2.3 Workload of clinicians and clinical dependency of each neonate

Subjective (perceived) workload was assessed using the paper version of the National Aeronautics and Space Administration task load index (NASA-TLX), a scale developed to measure domains in high-risk industries (Tubbs-Cooley et al., 2019). The NASA-TLX is primarily a measure of how clinicians (physicians and nurses) experience the situational demands of the healthcare work (the overall workload score from 0 (low) to 100 (high)) (Hart and Staveland, 1988). Covariables included duration of professional experiences (year), weekly working hours, and monthly number of shifts for each physician and nurse in the NICU.

We also collected infant acuity scores that corresponded to each infant-specific report of missed care. Infant acuity scores were determined as each admission in the NICU for determining an infant’s required nursing care within 24 h of admission. The score includes clinical indicators of nursing care intensity such as ventilation mode, feeding frequency and mode, number and type of infusions, and procedures. The range for each indicator varies depending on the number and type of items assessed, with higher scores indicating more intensive nursing care (1–5 levels).

### 2.4 Development, optimization, and validation of the machine learning-based model

In this study, we aimed to develop a machine learning-based model to predict the presence of medication errors. The outcome variable was binary, with the drug error either present or absent. The independent variables mainly include drug-related variables (total number of drugs, use of anti-infective drugs, systemic hormonal preparations, nervous system drugs, blood and blood-forming organ drugs, cardiovascular system drugs, alimentary tract and metabolism drugs, respiratory system drugs, and sensory organ drugs). Also, the workload parameters of the physicians and the nurses (duration of professional experience, weekly working hours, number of monthly shifts, and total NASA-TLX score) and demographic and clinical variables (such as gender, birth weight, gestational age, postnatal age, and APGAR score) were evaluated as independent variables. There was no missing value in the data; therefore, imputation was not performed.

We used a two-stage feature selection method. In the first stage, we performed univariate analysis to determine the candidate features that could be included in the final models using *IBM SPSS Statistics version 23*. We preferred univariate analysis because it allows for more conscious feature selection by enabling researchers to examine variables one by one. This facilitated the better understanding of the data (Chowdhury and Turin, 2020). The number of variables in this study was large enough to examine features one by one. Also, we identified highly correlated variables and selected those that were more clinically usable. Independent variables with *p* < 0.20 were selected after univariate analysis with a flexible approach.

After determining the candidate features using univariate analysis and reducing the size of the independent variable set, we performed feature selection in the second stage using machine learning methods with *R version 3.6.3*. At this stage, we used a multivariate feature selection method that examined all the independent features together. The data were randomly divided into train and test sets (70:30). The train set was used for developing the ML model using a 10-fold cross-validation method with R software (Kuhn, 2022). The test set was used to evaluate and validate model performance. Since train and test sets were randomly selected from the same data set, the characteristics of patients and the properties of variables were similar.

The *accuracy*, *sensitivity*, *specificity*, *positive predictive value* (PPV), *negative predictive value* (NPV), *F*_*1*_ *score*, and *area under ROC curve* (AUC) were used as performance measures in classification models to compare the performance of the models. A high-performance model requires these measurements of at least 0.70. We tried some of the ML methods that are frequently used in the literature. These methods were random forest (RF), elastic net, artificial neural network, and support vector machines with three different kernel functions (linear, radial basis, and polynomial). In most of the trials, the highest performance was provided by RF in terms of performance measures. Therefore, we decided to use RF for further analysis. Model performances were compared after hyperparameter optimization with tuneLength argument to avoid overfitting (Kuhn, 2012). Machine learning-based variable selection was made by selecting the most important variables for creating a webtool according to the importance plot. The models were trained after applying the z-transform to the quantitative features.

## 3 Results

### 3.1 Clinical characteristics

A total of 468 newborns were admitted to the 22-bed NICU of a tertiary referral hospital during the period of 17 months. Due to non-survival (n = 21%, 121%, and 4.5%) or lack of systemic medication treatment (n = 35% and 7.4%), 56 neonates were excluded. This led to the inclusion of 412 neonates in the study, of which 232 (56.3%) were males, 177 (43%) were born preterm [extremely preterm (<28 weeks): 7 (1.7%), very preterm (28–32 weeks): 52 (12.6%), moderate preterm (32–34 weeks): 16 (3.9%), and late preterm (34–37 weeks): 102 (24.8%)], and 172 (41.7%) had low birth weight (<2,500 g). The median (IQR) length of hospital stay (LOS) was 8 (11) days, and the median postnatal age (PNA) at admission was 1 (1) day.

During the study period, 11,908 medication orders (28.9 orders/patient) were generated, using the computerized physician order entry (CPOE) system for a total of 412 NICU patients (5.53 drugs/patient/day) who received 2,280 prescriptions over 32,925 patient days. The median (range) values for the total number of drugs and anti-infectives used during the hospitalization were 3 (0–29) and 2 (0–9), respectively. The most commonly prescribed medications were those for the anti-infective (38.82%), alimentary tract and metabolism (32.89%), and nervous system (8.07%) drugs. In total, 131 different medications were prescribed throughout the study period. Intravenous fluids (12.06%), gentamicin (8.03%), and ampicillin (7.81%) were the most commonly prescribed drugs.

### 3.2 NASA-TLX and infant acuity scores

A total of 18 pediatricians, four of whom were neonatologists, took part in the diagnosis, treatment, and care processes of the included neonates. The number of nurses who are constantly in the NICU was determined as 21. The median duration of professional experience for physicians was 1.16 years, and their mean NASA-TLX score was 65.16 points. In nurses, it was 8 years and 81 points, respectively. Based on the infant acuity score, 8% of the patients were classified as “unstable neonates, requiring complex critical care” and 21.6% as “requiring multi-system support within 24 h of admission” (Table 1).

**TABLE 1:** Infant acuity levels of the patients and the workload parameters of the clinicians.

Infant acuity level	n (%)
Continuing care	8 (1.9)
Requiring intermediate care	144 (35.0)
Requiring intensive care	138 (33.5)
Requiring multi-system support	89 (21.6)
Unstable, requiring complex critical care	33 (8.0)
Physicians	
Duration of professional experience (years), median (range)	1.16 (0.83–9)
Weekly working hours, median (range)	80 (60–110)
Number of monthly shifts, median (range)	10 (7–11)
NASA-TLX subscales, median (range)	
Mental demand	90 (30–100)
Physical demand	70 (35–100)
Temporal demand	75 (20–100)
Effort	15 (5–80)
Performance	80 (60–100)
Frustration level	70 (5–100)
Total NASA-TLX score, median (range)	65.16 (48.66–90.00)
Nurses	
Duration of professional experience (years), median (range)	8 (1–17)
Weekly working hours, median (range)	40 (7–52)
Number of monthly shifts, median (range)	7 (0–10)
NASA-TLX subscales, median (range)	
Mental demand	100 (75–100)
Physical demand	95 (55–100)
Temporal demand	95 (5–100)
Effort	15 (5–100)
Performance	95 (10–100)
Frustration level	90 (15–100)
Total NASA-TLX score, median (range)	81 (40–100)

NASA-TLX, National Aeronautics and Space Administration task load index.

### 3.3 Characteristics of the medication errors

Depending on the LOS, the number of medication orders varied between 1 and 131. At least one type of MEs was detected in 257 (62.4%) of the patients. The median (range) number of ME types observed in these patients were 2 (1–8), while 93 (22.6%) patients had one ME, 61 (14.8%) had two ME, 49 (11.9%) had three ME, and 54 (13.0%) had at least four ME types. When examining how many determined ME cumulative days for each patient, it was observed that the median (range) was 6 (0–275) days. When the number of MEs per day during the hospitalization of the patients was examined, the median (range) was 0.50 (0–11.50) MEs/day.

When the types of MEs originating from physicians and nurses were analyzed separately, at least one type of ME originating from a physician was found in 174 (42.2%) of the patients, while at least one type of ME originating from a nurse was found in 235 (57.0%) of the patients. When the ME types were examined separately, the most common MEs were determined as the physician-related wrong infusion rate (25.24%) and nurse-related wrong administration time (52.66%) according to the medication orders. When the total exposure day of the patients to these MEs (how long did detected ME lasts) was examined, physician-related wrong dose (507 days, 8.52%) and nurse-related wrong administration time (3,212 days, 53.95%) were the most common types of ME (Table 2).

**TABLE 2:** Distribution of the number of patients, duration of exposure, and total patient days for each medication error.

	Type of ME	Number of patients, n (%)	Total exposure day (range)	Total exposure day/total patient day (%)
Physicians	Prescription			
	Wrong drug	46 (11.16)	154 (0–23)	2.59
	Wrong unit	11 (2.67)	53 (0–26)	0.89
	Wrong dose	78 (18.93)	507 (0–33)	8.52
	Wrong dose interval	17 (4.12)	136 (0–21)	2.29
	Wrong infusion rate	104 (25.24)	145 (0–10)	2.44
	Wrong diluent	17 (4.12)	82 (0–8)	1.37
	Monitoring			
	Physical	3 (0.72)	14 (0–8)	0.23
	Vital	1 (0.24)	7 (0–6)	0.11
	Laboratory	10 (2.40)	56 (0–15)	0.94
	TDM	8 (1.94)	28 (0–12)	0.47
Nurses	Preparation			
	Wrong drug	14 (3.39)	149 (0–60)	2.50
	Wrong occupational safety	8 (1.94)	54 (0–15)	0.90
	Wrong storage	23 (5.58)	65 (0–8)	1.09
	Administration			
	Dose omission	1 (0.24)	1 (0–1)	0.01
	Extra dose	0 (0)	0	0
	Wrong time	217 (52.66)	3,212 (0–140)	53.95
	Wrong infusion	33 (8.00)	157 (0–18)	2.64
	Wrong technique	33 (8.00)	296 (0–48)	4.98
Total		257 (62.40)	5,116	85.92

ME, medication error; TDM, therapeutic drug monitoring.

**FIGURE 2:**
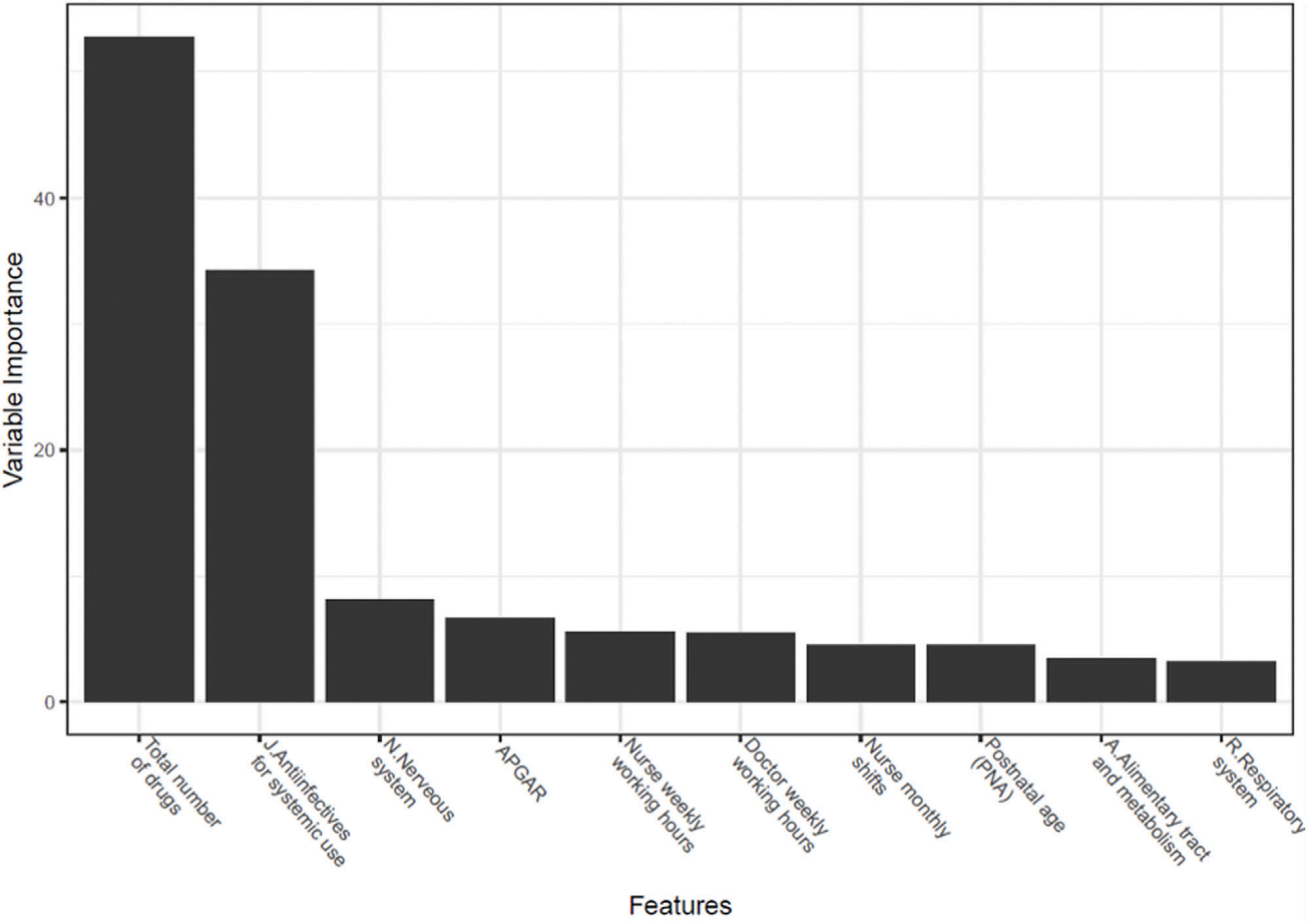
Variable importance plot (%) used to predict the presence of medication errors.

According to the univariate analysis, there was a significant relationship between drug error and gestational age, birth weight, use of drugs (anti-infectives for systemic use, systemic hormonal preparations, nervous system, alimentary tract and metabolism, cardiovascular system, respiratory system, and sensory organs), postnatal age, 5-min APGAR score, the total number of drugs, and the number of nurse monthly shifts (*p* < 0.05). The *p*-values for the relationships between the drug error with weekly working hours of physicians and weekly working hours of nurses were both less than 0.20. These variables were used in the classification model. The parameters that have the highest correlation with the occurrence of ME and included in the model were: total number of drugs, anti-infective drugs, nervous system drugs, 5-min APGAR score, postnatal age, alimentary tract and metabolism drugs, and respiratory system drugs as patient-related parameters and weekly working hours of nurses, weekly working hours of physicians, and number of nurses’ monthly shifts as care provider-related parameters. It was observed that the most important variables in predicting the presence of ME with machine learning algorithms were the total number of drugs and the prescription of anti-infective drugs (Figure 2). It was determined that the obtained model showed a high performance in predicting the presence of ME. Test set performance measures of the model were calculated. The prevalence (the ratio of having a drug error) was close between the train and test set (64% for the train set and 59% for the test set). The performance measures were calculated as follows: *accuracy* 0.919 (95% CI 0.858–0.956), *sensitivity* 0.918 (95% CI 0.844–0.964), *specificity* 0.922 (95% CI 0.829–0.973), PPV 0.944 (95% CI 0.884–0.974), NPV 0.887 (95% CI) 0.804–0.937), AUC 0.920 (95% CI 0.876–0.970), and *F*_*1*_ *score* 0.931. A higher AUC indicated that the model predicting the presence of physician- or nurse-related MEs correctly classified 92.0% of the patients. This prediction model is available to clinicians as a free, user-friendly, and registration-free web-tool (http://softmed.hacettepe.edu.tr/NEO-DEER_Medication_Error/). Additionally, the codes are available at https://github.com/mervekasikci/NEO-DEER_Medication_Error-/tree/main.

## 4 Discussion

The incidence of MEs in hospitalized neonates is higher than that in other populations (Kaushal et al., 2001; Alghamdi et al., 2019; Alghamdi et al., 2021). This situation relates to factors specific to the neonatal intensive care environment such as differences in clinician’s workload and the diversity in pharmacotherapy, as well as patient-related factors such as rapidly physiological changes (body weight, volume of distribution, renal function, etc.) and subsequent variability in dosing.

In our study, at least one ME was found in 62.4% of the patients by evaluating 14.45 medication orders/patient. Leopoldino et al. (2019a) reported a similar rate (59.8%) in a single NICU in a teaching hospital in Brazil. Failure to administer drugs at the right time (52.6%) resulted in a higher-than-expected rate of nurse-related MEs. In other words, during the study period, there was variability between the time of the drugs prescribed by the physicians and the time of administration by the nurses. The primary motivation for documenting these errors was not to recognize them as potentially high-risk MEs for the patient. The main aims were to examine the inconsistency in prescribing and practice habits between physicians and nurses and to provide information about the procedure of the CPOE system in the NICU. Although the CPOE system is integrated into the hospital, it is reported in the literature that not administering drugs by the nurses at the time prescribed increases the risk of wrong dose, omission, or duplication (FitzHenry et al., 2007).

Artificial intelligence and machine learning tools are increasingly used in healthcare for screening, diagnosis, pharmacovigilance, outcome prediction, and reducing medical errors as a precision concept. There are many novel studies in the literature showing that using these tools for the integration of clinical data will pave the way for precision medicine in neonatology (Bate and Luo, 2022; Cherkas et al., 2022; Zhao et al., 2022).

In the current literature, reported risk factors for MEs are gestational age (<3 weeks), total number of drugs (>3), prescription of anti-infective and intravenous drugs, APGAR score (<7), length of hospital stay (>7 days), neurological, renal, and cardiovascular diseases, workload of clinicians, and lack of pharmacotherapy education (Rashed et al., 2014; Zhang et al., 2017; Leopoldino et al., 2019b; Palmero et al., 2019; Bharathi et al., 2020). In our study, almost all these variables were taken into consideration, and an ME prediction model was designed. In addition, it has been confirmed that the required nursing time per patient per day, which is an independent indicator of workload, was a risk factor in the occurrence of MEs (Figure 2) (Morriss et al., 2009). According to another study, the risk factors for MEs were workload, inadequate guidelines, and the lack of design in the systems and protocols (Alghamdi et al., 2021). According to a model developed by Leviatan et al. (2021), physicians working two or three consecutive shifts and physicians with less prescribing experience had a higher rate of MEs (*p* < 0.01). In our study, in the high-performance model that predicts the presence of MEs, the weekly working hours of physicians were only found to be the sixth most important variable (AUC: 0.920). Similar studies have also been carried out for nurses.

Tubbs-Cooley et al. (2019) showed that as the infant acuity level, which is an indicator of the complexity of treatment and care, increases in NICU patients, the risk of not being able to control all of the six “rights” (right patient, medication, dose, time, route, and documentation) that nurses should pay attention to increased by 1.05 (95% CI 1.02–1.07) times (*p* < 0.004). In our study, the infant acuity level and NASA-TLX score were not found to be a significant parameter in predicting the presence of MEs. However, weekly working hours and monthly shifts, which indicated that nurses worked more, were found to be important parameters in predicting the presence of MEs.

In a study in which each ME in NICU patients (n = 410) was classified and predicted as appropriate–inappropriate, the *F*_*1*_ *score* indicating model performance was found to be 0.13 (Hogue et al., 2021). In our study, in which a total of 5,954 medication orders specifically for the NICU were examined, the prediction performance of the model obtained with patients categorized as ME detected or not detected was found to be much higher (*F*_*1*_ *score*: 0.931). In ensuring the accuracy of this model, it is estimated that the prospective real-life study design, the study in a specific unit, and the selection of the correct clinical and demographic parameters throughout the study were effective.

As is known, automatic warning systems integrated into CPOE systems provide only theoretical information and warnings about the prescribed drug, without showing a patient-centered approach (Schiff et al., 2017). It is known that the warnings that occur in this situation cause alert fatigue in clinicians and 90%–96% of them are ignored by them. A study predicting physician response to more than 6,000 prescription alerts also demonstrated that theoretical warnings were ignored with the high-performance model (*accuracy*: 0.850, AUC: 0.940) (Poly et al., 2020). With our machine learning-based clinical decision support tool, it is expected to predict whether MEs will occur with a newborn-centered approach without causing alert fatigue. Such approaches can support precision pharmacovigilance and facilitate implementation in clinical care.

Although the targeted sample size was reached and this is a prospective study to obtain real-life data in the study population, this study has still some limitations. The data obtained from a single center limit the heterogeneity of the data pool. A lack of generalizability to other populations and the need for further validation are other limitations acknowledged by the authors. In addition, since it reduced the performance of the model, the type of ME was not included as an output variable.

There were no confounding variables such as clinical pharmacist interventions that could affect outcomes. It is considered to include independent variables (output) in future studies instead of additional clinical parameters to be included in the study as dependent variables (input). It is suggested to focus on how long the MEs persist, the root cause of the MEs (medication discrepancies, near-miss errors, potential harms, etc.), alert fatigue in clinicians, and the integration of the system into clinical pharmacy interventions.

This is the first developed and validated model to predict the presence of MEs using work environment and pharmacotherapy parameters with high-performance ML algorithms in NICU patients. Taking these limitations into account, this approach and the current model hold the promise of implementation of precision screening to prevent MEs in neonates. ME prevention can be optimized by identifying patients who require targeted clinical pharmacy services. As clinical pharmacy practices are not available in every NICU and capacity is limited, a prediction model can be used to compensate for this increasing workload and decreasing the number of qualified healthcare providers.

## Data Availability

The raw data supporting the conclusion of this article will be made available by the authors, without undue reservation.
